# COVID-19: Characteristics and Therapeutics

**DOI:** 10.3390/cells10020206

**Published:** 2021-01-21

**Authors:** Rameswari Chilamakuri, Saurabh Agarwal

**Affiliations:** Department of Pharmaceutical Sciences, College of Pharmacy and Health Sciences, St. John’s University, 8000 Utopia Parkway, Queens, NY 11439, USA; rameswari.chilamakuri19@my.stjohns.edu

**Keywords:** COVID-19, SARS-CoV-2, coronavirus, pandemic, vaccine, therapeutics, epidemiology, spike protein

## Abstract

Novel coronavirus (COVID-19 or 2019-nCoV or SARS-CoV-2), which suddenly emerged in December 2019 is still haunting the entire human race and has affected not only the healthcare system but also the global socioeconomic balances. COVID-19 was quickly designated as a global pandemic by the World Health Organization as there have been about 98.0 million confirmed cases and about 2.0 million confirmed deaths, as of January 2021. Although, our understanding of COVID-19 has significantly increased since its outbreak, and multiple treatment approaches and pharmacological interventions have been tested or are currently under development to mitigate its risk-factors. Recently, some vaccine candidates showed around 95% clinical efficacy, and now receiving emergency use approvals in different countries. US FDA recently approved BNT162 and mRNA-1273 vaccines developed by Pfizer/BioNTech and Moderna Inc. for emergency use and vaccination in the USA. In this review, we present a succinct overview of the SARS-CoV-2 virus structure, molecular mechanisms of infection, COVID-19 epidemiology, diagnosis, and clinical manifestations. We also systematize different treatment strategies and clinical trials initiated after the pandemic outbreak, based on viral infection and replication mechanisms. Additionally, we reviewed the novel pharmacological intervention approaches and vaccine development strategies against COVID-19. We speculate that the current pandemic emergency will trigger detailed studies of coronaviruses, their mechanism of infection, development of systematic drug repurposing approaches, and novel drug discoveries for current and future pandemic outbreaks.

## 1. Introduction

Novel pneumonia caused by coronavirus was reported in the Chinese city of Wuhan in December 2019. The virus was then identified and named as a severe acute respiratory syndrome coronavirus (SARS-CoV-2) or COVID-19. COVID-19 is an acronym that stands for coronavirus disease of 2019 and is given by the World Health Organization (WHO). The International Committee on Taxonomy of Viruses (ICTV) suggested that this novel coronavirus was named SARS-CoV-2 due to the phylogenetic and taxonomic analysis of this novel coronavirus [1,2]. According to the Johns Hopkins Coronavirus resource center (coronavirus.jhu.edu), as of January 2021, a total of about 98.0 million cases along with 2.0 million deaths related to COVID-19 have been confirmed worldwide. This highlights the severity of COVID-19 infection, and also the nonavailability of effective therapy to date.

According to the Center for Disease Control and Prevention (CDC; https://www.cdc.gov/coronavirus/types.html), three out of the seven coronaviruses that epidemically outbreak in humans include SARS-CoV in 2002 (severe acute respiratory syndrome or SARS), MERS-CoV in 2012 (Middle East respiratory syndrome or MERS), and now SARS-CoV-2 (the current pandemic known as COVID-19) [3,4]. The other four coronavirus stains, such as 229E, NL63, OC43, and HKU1, are also known to infect humans. A wide distribution, human–animal transition, and frequent recombination of the genetic material of the coronaviruses are likely causes of the frequent and periodic appearances in humans [3,4,5]. The typical symptoms of a patient who has been infected with COVID-19 are fever, dry cough, dyspnea, myalgia, fatigue, normal or decreased leukocyte counts, and radiographic evidence of pneumonia. Some COVID-19 patients have also reported radiological ground-glass lung changes, lymphopenia, and thrombocytopenia symptoms [3,6,7].

Detailed studies of this novel coronavirus, its mechanism of infection, and replication are needed for developing effective therapeutic approaches and vaccination-mediated immunological memory, to effectively fight the current and potential future pandemics. In this review, we present a systematic overview of COVID-19 epidemiology, structure, molecular mechanisms of infection, pathophysiology, diagnosis, and different developmental therapeutic and vaccine approaches underway.

## 2. COVID-19 Epidemiology

To date, it has been found that all ages are susceptible to COVID-19 infection. The world map of COVID-19 mediated infections and deaths showed that no country, race, ethnicity, or religion is spared from this virus (https://coronavirus.jhu.edu/map.html). The possible transmission route of this novel coronavirus is person-to-person, which includes contact transmission by contacting the nasal, oral, and eye mucosal secretions of the infected patient, as well as direct transmission by droplet inhalation when the patient coughs or sneezes [8,9]. Although there are no known ophthalmological symptoms, eye exposure may provide a productive way for the virus to enter the body. Recent evidence has also suggested that COVID-19 can be transmitted to unborn babies through the placenta in utero [10]. Studies have shown higher viral loads in the throat, while no significant difference in viral burden has been observed when comparing symptomatic and asymptomatic cases [11]. It is also reported that droplets from sneezing or coughing can spread up to 6 feet, emphasizing the 6 feet social distancing criteria [12]. The virus can deposit on many surfaces and can survive for days under favorable conditions depending on the particular surface. The incubation period for COVID-19 is from 3 to 14 days, depending upon the patient’s immunological conditions [13].

## 3. SARS-CoV-2 Virus Structure and Integration

**Figure 1:**
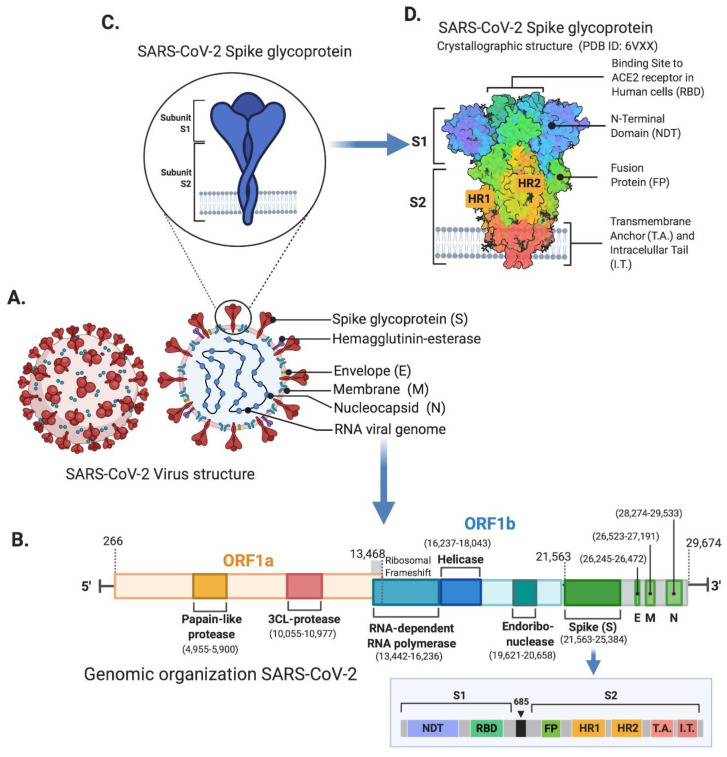
Structure and genomic organization of SARS-CoV-2. (**A**) Schematic representation of SARS-CoV-2 virus structure and the positions of spike glycoprotein, hemagglutinin-esterase, envelope, membrane, nucleocapsid, and RNA viral genome. (**B**) Genomic organization of SARS-CoV-2 representing ORF1a, ORF1B which encode for nonstructural proteins such as papain-like protease, 3CL-protease, RNA-dependent RNA polymerase, helicase, and endoribonuclease. Genes coding for spike (S), envelope (E), membrane (M), and nucleocapsid (N) proteins are also displayed. Ribosomal frameshift location between ORF1 and ORF2 is shown at the junction of ORF1/2. Genomic positions are shown with dashed lines followed by nucleotide position number in RNA viral genome. The box highlights the genomic organization of spike (S) gene showing distinct S1 and S2 subunits coding segments. (**C**) Schematic magnified representation of SARS-CoV-2 spike glycoprotein showing S1 and S2 subunits. (**D**) Crystallographic structure of SARS-CoV-2 spike glycoprotein adapted from PDB ID:6VXX. Receptor binding domain (RBD) representing ACE2 receptor binding site in human cells, N-terminal domain (NTD), fusion protein (FP), transmembrane anchor (T.A.), and intracellular tail (I.T.) protein domains are displayed.

SARS-CoV-2 belongs to the Betacoronavirus genus and is a member of the *Coronavirinae* family [14]. The virus particles are spherical or pleomorphic in shape, with a diameter of about 60–140 nm. Coronaviruses have one of the largest single-strand RNA genomes with 27–32 kilobases (kb) (Figure 1) [15]. Some of the coronaviruses encode for the hemagglutinin-esterase protein, 3a/b protein, and 4a/b protein on their surface [15,16,17,18,19]. The genome organization of SARS-CoV-2 is similar to other coronaviruses, which is composed of mainly the open reading frames (ORFs). Roughly 67% of the genome encodes by the ORF1a/b and it encodes for 16 nonstructural polyproteins (nsp1-16), while the remaining 33% encodes for accessory proteins and structural proteins. ORF1a and ORF1b contain a frameshift which produces two polypeptides, pp1a and pp1ab. Papain-like protease (PLpro) or chymotrypsin-like protease (3CLpro), process these two polypeptides into 16 nsps (Figure 1B) [20]. SARS-CoV-2 encodes for at least four major structural proteins that includes spike protein (S), membrane protein (M), an envelope protein (E), and nucleocapsid protein (N). These structural proteins are encoded by S, M, E, N genes at ORFs 10 and 11 on the one-third of the genome near the 3′-end (Figure 1A,B) [21]. These mature structural proteins are responsible for viral maintenance and replication [17]. Most of the probes and primers used to detect the SARS-CoV-2 are constructed against the genetic targets of ORF1ab and the N gene region [22].

**Figure 2:**
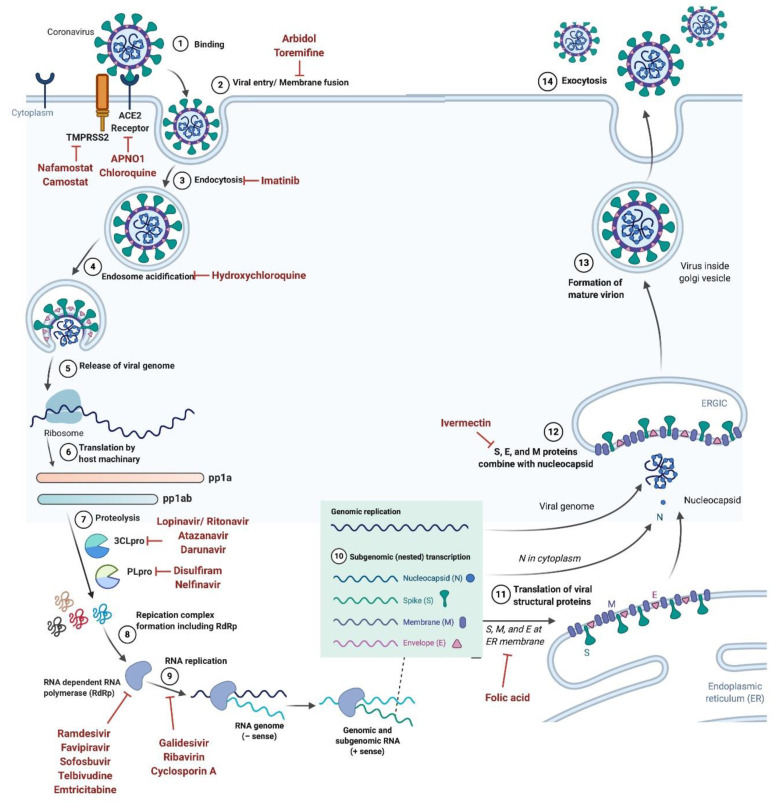
Schematic representation of SARS-CoV-2 virus life cycle. Drugs targeting different steps of coronavirus entry and lifecycle in human cells are also shown.

Once the virus enters into a host cell, the synthesis of structural and accessory proteins begins with transcription and translation processes. The synthesis of the new viral RNA genome occurs with the help of RNA-dependent RNA polymerase, which utilizes the negative stand template (Figure 2) [15,23]. The binding affinity of SARS-CoV-2 for the angiotensin-converting enzyme 2 (ACE2) receptor is higher than other SARSs, which in turn facilitates the rapid transmission of SARS-CoV-2 [15,23,24]. The M protein is the most abundant structural glycoprotein and is responsible for the transport of nutrients across the cell membrane while giving shape to the virus particle [25]. The S or spike protein is a type I membrane glycoprotein which constitutes virus peplomers. The N protein aids in binding the viral RNA genome while maintaining RNA stability [26]. The E protein plays an important role in viral release as well as assembly during pathogenesis (Figure 1 and Figure 2) [27]. The analysis of the whole genome sequence of SARS-CoV-2 shows that it shares 85-95% sequence similarity with SARS-CoV, indicating that SARS-CoV-2 is more compatible with SARS-CoV [27].

## 4. SARS-CoV-2 Virus Receptor Mechanism

As mentioned above, spike (S) protein, which is located on the surface of SARS-CoV-2 is vital for infection and pathogenesis. The entry of SARS-CoV-2 into a host cell is mediated by the S protein, which ultimately gives coronaviruses a crown-like appearance as they form spikes on their surface (Figure 1C,D). The S protein consists of three subunits, the ectodomain, a single-pass transmembrane anchor, and an intracellular C-terminal tail [28]. The ectodomain can be further divided into a receptor-binding S1 subunit and a membrane-fusion S2 subunit (Figure 1C,D) [29]. The SARS-CoV-2 virus enters the host cell through the interaction of the receptor-binding S1 subunit with the ACE2 receptor on the host cell surface [30]. Human ACE2 receptors are expressed in almost all tissues, and they are most abundant in the lungs, kidneys, brain stem, adipose tissue, heart, vasculature, stomach, liver, as well as the nasal and oral mucosa [31]. The S2 subunit fuses the host and viral membranes, while facilitating the entry of the viral genome into host cells [32]. This process requires S protein priming by host cell proteases, which leads to S protein cleavage at the S1–S2 boundary (Figure 1D and Figure 2). Recent reports have showed that SARS-CoV-2 uses ACE2 for entry, while utilizing the transmembrane protease, serine 2 (TMPRSS2), and endosomal cysteine proteases cathepsin B and L (CatB/L), for S protein priming (Figure 2) [33]. Going forward, knowledge about the receptor recognition and interaction mechanisms will be critical in identifying effective therapeutic targets.

## 5. Clinical Manifestations

The complete clinical manifestations of SARS-CoV-2 are not yet clear, but the symptoms range from asymptomatic or mild to severe. Both elderly and young patients can succumb to death depending on their underlying health conditions such as cardiovascular diseases, kidney damage, liver dysfunction, diabetes, Parkinson’s disease, and cancer [34,35]. Healthy individuals may recover from the viral infection within 2–4 weeks of treatment [36]. As of January 2021, recovery has been observed in about 70 million confirmed coronavirus cases and were then found to be negative for the virus at diagnosis.

The most commonly reported symptoms in COVID-19 patients are fever or chills, headache, muscle or body aches, dry cough, myalgia or fatigue, pneumonia, and complicated dyspnea. The less commonly reported symptoms include tastelessness, diarrhea, hemoptysis, runny nose, liver damage, kidney damage, nausea, and vomiting. In most of the symptomatic patients, symptoms start from 2 to 14 days after viral exposure [36,37].

## 6. Diagnosis

### 6.1. Nucleic Acid Detection-Based Assays

The current method for the clinical diagnosis of SARS-CoV-2 is nucleic acid detection in nasopharyngeal and oropharyngeal samples by a reverse transcription-quantitative polymerase chain reaction (RT-qPCR) method [38]. RP primers, 2019-nCoV_N1, 2019-nCoV_N2, and 2019-nCoV_N3 primers and probes which target the nucleocapsid (N) gene and human RNase P gene were designed and are currently in use for universal detection of SARS-like coronaviruses and 2019-nCoV [39]. Although RT-qPCR is very specific, on occasion it can give false negative results due to sample contamination or technical faults. These issues cannot be ignored due to the severe consequences of a missed diagnosis, especially in the case of COVID-19 [40,41]. Additionally, RT-qPCR is a time-consuming protocol that requires the extraction of RNA, needs well trained laboratory technicians, and increases the risk of the exposer to viral droplets or samples [3,42,43]. To avoid some of these limitations and provide quick diagnosis turn-around time, other detection methods such as reverse transcription loop-mediated isothermal amplification (RT-LAMP), transcription-mediated amplification (TMA), CRISPR-based assays, rolling circle amplification, and microarray hybridization assays were developed and are currently in use [44].

### 6.2. Serological and Immunological Assays

Several diagnostic tests for detecting those who are either currently infected or have been previously infected with SARS-CoV-2 have been developed based on blood sample analysis which detects the presence of IgM and IgG antibodies [45]. IgM antibodies first become detectable in serum during the initial few weeks post-infection, and then the isotype switches to IgG. Therefore, IgM provides an indication of early-stage infection, while IgG indicates a current or prior infection [46]. This test plays an important role in the epidemiology and vaccine development for SARS-CoV-2 and it provides an assessment of both short- and long-term antibody response, antibody abundance, and diversity. These tests are easy to perform, provide a rapid response, and are therefore a high-throughput method for diagnosing viral infections.

Developing serological tests often relies on finding suitable viral antigens or recombinant proteins to capture host antibodies. For SARS-CoV-2, spike (S) glycoprotein and nucleocapsid (N) phosphoprotein which are abundantly expressed on SARS-CoV-2 virus surface serves as the primary antigenic targets against which antibodies are detected [47,48]. Among the S glycoprotein S1 subunit in SARS-CoV-2 showed least overlap with the other coronaviruses, making S1 and N proteins the most suitable antigens for COVID-19 serological tests [49]. Different types of antigen detection methods are used to determine the antibody functionality and immune response.

#### 6.2.1. Binding Antibody Detection Methods

Purified SARS-CoV-2 proteins are used in the binding antibody detection techniques. These tests can be performed rapidly using specific reagents. Antibody detection methods have great advantages, including rapid response, low cost, and portability compared to molecular detection methods. However, these tests are less reliable and often require confirmation with molecular detection methods such as RT-qPCR, which takes additional time and costs [43,44,50,51,52]. Tests that detect antigen binding antibodies mostly fall under two broad categories.

Point of Care Tests (POC)

These tests detect either total antibody or IgG and IgM in the saliva, plasma, serum, or whole blood samples. POC tests can be performed quickly on blood samples obtained from finger sticks or nasopharyngeal and oropharyngeal swabs [53]. In response to the COVID-19 pandemic and shortages of laboratory-based molecular testing capacities and reagents, multiple diagnostic test manufacturers have developed rapid and easy-to-use devices to facilitate testing outside of laboratories. FDA authorized Abbott Alinity I SARS-CoV-2 IgG, Access Bio CareStart COVID-19 IgM/IgG, and Beckman Coulter Access SARS-CoV-2 IgG kits for emergency SARS-CoV-2 testing in schools, hospitals, and testing centers. Most of these tests show the final results as colored lines for naked eye detection with a control line confirming test reliability. Colored test lines indicate the presence of target antibodies. These tests are rapid, cost-effective, and do not require specialized training. Although, validity and efficacy of these tests are very low, and often need a secondary validation using RT-qPCR based method.

Laboratory Tests

These tests include enzyme-linked immunosorbent assay (ELISA), chemiluminescence immunoassay (CIA), and lateral flow immunoassay (LFI). Specific instruments, reagents, or well-trained technicians are needed for these assays [51]. ELISA is a high sensitivity and throughput diagnostic method, which uses multiwell plates coated with viral proteins. Blood, plasma, or serum samples from patients are diagnosed for antibody capture and detection. The analytical sensitivity of these tests is very high with rapid detection time, but require trained technicians and often require secondary test result validation [54].

#### 6.2.2. Neutralizing Antibody Detection

Neutralizing antibody detection method requires highly sophisticated biosafety laboratories depending on the type of SARS-CoV-2 virus. These tests involve incubating serum or plasma with live virus followed by infection and incubation with cells. Different types of neutralizing antibody tests include virus neutralization tests (VNT), which is a gold standard to detect active antibodies against live virus. The test results are typically read out by microscopy for viral cytopathic effect where neutralizing antibodies would block virus replication and let cells grow. Similarly, plaque reduction neutralization test (PRNT) and focus reduction neutralization test (FRNT) were developed. Although highly specific, VNT is time-intensive and requires specialty laboratory facilities. Therefore, these tests are primarily used for vaccine and therapeutic developments.

#### 6.2.3. Antigen Detection Methods

These assays detect the presence of viral antigens via conventional immunocapture format. SARS-CoV-2 viral antigen N protein can be detected when the virus is actively replicating, which makes this assay type highly specific. Several rapid test kits are under development for antigen detection methods for SARS-CoV-2. These tests require high antigen presence, sophisticated equipment, and trained laboratory technicians [55].

### 6.3. Chest Computed Tomography (CT) Scan

CT scans are widely used tests and are more specific than RT-qPCR in diagnosing SARS-CoV-2 infection [56]. SARS-CoV-2 infected patients experience symptoms typical for pneumonia during the early stages of infection [57]. In some patients both nasopharyngeal and oropharyngeal swabs, as well as stool samples, tested positive for SARS-CoV-2, while blood samples tested negative [58]. In these cases, chest CT findings provide confirming diagnosis associated with COVID-19 pneumonia that includes bilateral parenchymal ground-glass opacities (80%) and unilateral lung with subpleural lesions (20%) [59,60].

## 7. Potential Treatment Strategies

As of today, there is no clinically proven and specific antiviral drug available for treating the SARS-CoV-2 infection [61]. Most treatment trials are based on molecular mechanisms and genomic organization of SARS-CoV-2. There are several existing antiviral agents that could potentially be repurposed or developed into effective interventions for this novel coronavirus. Some of these approaches are detailed below.

### 7.1. Inhibiting the RNA-Dependent RNA Polymerase

#### 7.1.1. Remdesivir

Remdesivir or GS-5734, is a broad-spectrum, antiviral, phosphoramidate pro-drug [62]. In the body it is converted to GS-441524, a ribonucleotide analog. It is currently in clinical trials for the treatment of the Ebola viral infection [63,64]. Remdesivir is an adenosine analog that interferes with the RNA-dependent RNA polymerase enzyme, which incorporates ribonucleotides into nascent viral RNA chains (Figure 2). Due to this mechanism, Remdesivir confuses viral RNA-dependent RNA polymerase and delays, or prematurely terminates, RNA chains which in turn inhibits viral RNA production and replication of EBOV [65,66]. Viruses with mutated RNA polymerase often develop partial resistance against Remdesivir [67,68]. Recent research has demonstrated that Remdesivir is a potential therapeutic for the treatment of SARS-CoV-2. According to The New England Journal of Medicine [11], the first case in the United States (reported in Washington State) was treated with an intravenous administration of Remdesivir which mitigated symptoms, and did not cause any side-effects [11]. However, prior clinical trials in Ebola reported side effects such as liver inflammation, nausea, sweating, shivering, and low blood pressure [69]. Remdesivir is currently in clinical trial evaluation for the treatment of COVID-19 in various countries including France (NCT04365725), USA (NCT04431453), Canada (NCT04330690), Egypt (NCT04345419) (Table 1). Remdesivir is currently in Phase 3 clinical trials in the USA, but on May 1st, 2020, the FDA issued an emergency use authorization for Remdesivir to treat COVID-19 patients [70,71,72].

#### 7.1.2. Favipiravir

Favipiravir, also known as Avigan or T-705, is a potential antiviral, and anti-influenza drug approved in Japan for the treatment for influenza A, B, and C viruses, including oseltamivir-resistant strains [73]. Favipiravir is under clinical trials for the treatment of the Ebola virus infection and has been reported to be effective against low to moderate levels of Ebola infections, but not for high-risk groups [74,75]. Favipiravir is a pro-drug that undergoes intracellular metabolism by the human hypoxanthine guanine phosphoribosyltransferase enzyme which then yields the active favipiravir-ribofuranosyl-5′-triphosphate. Favipiravir is a nucleoside analog that inhibits the viral RNA-dependent-RNA-polymerase enzyme and prevents the viral RNA replication process (Figure 2) [76]. Previous studies have shown that Favipiravir not only reduced the viral load in the upper respiratory tract, but also reduced the viral load in the lungs [77]. Favipiravir is currently under clinical trials for the treatment of novel SARS-CoV-2 (Table 1) [70,73,78].

#### 7.1.3. Galidesivir

Galidesivir, also known as BCX4430 or Immucillin-A, is a potential antiviral drug, and was originally developed to treat hepatitis C [79,80]. Galidesivir prevents the replication and transcription of the viral genome. Galidesivir binds to the viral enzyme’s active sites and incorporates itself into viral RNA strands, leading to chain termination (Figure 2) [81]. It is currently under clinical trials for the treatment of the Ebola virus infection, Filovirus Infections, Marburg Virus Disease, and for novel SARS-CoV-2 (NCT03891420).

#### 7.1.4. Ribavirin

Ribavirin, also known as Tribavirin, is a broad-spectrum antiviral drug [82]. Ribavirin is a guanosine analog and inhibits guanosine monophosphate (GMP) synthesis as a competitive inhibitor of the inosine monophosphate dehydrogenase enzyme. Inosine monophosphate dehydrogenase catalyzes the rate-limiting step where inosine 5′-monophosphate is converted to xanthine monophosphate during GMP synthesis. GMP is then further converted into guanosine triphosphate. By inhibiting this pathway, Ribavirin inhibits the viral mRNA synthesis, viral protein synthesis, as well as replication of RNA and DNA viruses. Ribavirin is approved for the treatment of hepatitis C infections and also for viral hemorrhagic fevers [83,84,85]. Ribavirin also causes mutations in the targeted viral RNA, premature termination of nascent RNA, and increases mutagenesis by producing defective virions [86]. Due to these properties, Ribavirin is currently in clinical trials for the treatment of SARS-CoV-2 (Table 1).

#### 7.1.5. Sofosbuvir

Sofosbuvir, also known as Sovaldi, is a direct-acting antiviral drug used to treat hepatitis C in combination with other antiviral drugs such as Ribavirin, Velpatasvir, and Elbasvir [87,88,89,90]. Sofosbuvir is a pro-drug that undergoes hepatic metabolism to form the active antiviral compound 2′-deoxy-2′-α-fluoro-β-C-methyluridine-5′-triphosphate [91]. This compound acts as a nucleotide analog inhibitor for the RNA-dependent RNA polymerase enzyme, which is vital for hepatitis C viral RNA synthesis and replication. This drug is also reported to inhibit the synthesis of the Zika virus by inhibiting pTBK1 localization and mitosis in human neuroepithelial stem cells. Sofosbuvir is currently in Phase 2/3/4 clinical trials in combination with other antiviral drugs for the treatment of SARS-CoV-2 (Table 1) [90,92].

### 7.2. Viral Protease Inhibitors

#### 7.2.1. Lopinavir/Ritonavir

Lopinavir and Ritonavir are anti-retroviral protease inhibitors which are used alone or in combination with other anti-retroviral agents to treat the human immunodeficiency virus (HIV) infection. The aspartyl protease enzyme, which is encoded by the pol gene of HIV, cleaves the precursor polypeptides in HIV and plays an essential role in viral replication [85,93]. Lopinavir and Ritonavir both inhibit the HIV protease enzyme by blocking its active site. Although coronaviruses encode a different enzymatic class of protease, the cysteine protease, but research studies have shown that Lopinavir and Ritonavir are effective against the SARS and MERS viruses (Figure 2) [94]. Clinical trials using both Lopinavir and Ritonavir on SARS-CoV-2 infected patients have exhibited little to moderate benefits for improving the clinical outcome (Table 1).

#### 7.2.2. Nelfinavir

Nelfinavir, also known as Viracept, is an antiviral drug used to treat HIV infections as a first-line therapy in combination with other HIV medications. Nelfinavir inhibits HIV-1 and HIV-2 retroviral proteases, which are vital for both viral replication within the cell and also for the release of mature viral particles from an infected cell [95]. The underlying mechanism for how Nelfinavir inhibits SARS-CoV-2 replication remains unknown. The main proteases of SARS-CoV-2 play a vital role both during and after infection as well as in viral replication, therefore, the effect of Nelfinavir on the main protease activity of SARS-CoV-2 should be investigated. In vitro studies have shown that Nelfinavir inhibits the replication of SARS-CoV-2 and is a promising drug for treating the coronavirus infection [96].

#### 7.2.3. Atazanavir

Atazanavir, also known as Reyataz, is an anti-retroviral drug that belongs to the protease inhibitor class and is used to treat HIV infections. Atazanavir binds to the active sites of the HIV-1 protease enzyme in infected cells, and selectively inhibits the processing of viral Gag and Gag-Pol polyproteins. Atazanavir inhibits the SARS-CoV-2 proteases enzyme and prevents the formation of mature viral particles in combination with other inhibitors [97]. Atazanavir is currently in Phase 2 clinical trials for the treatment of SARS-CoV-2 in combination with Nitazoxanide, Ritonavir, Dexamethasone, and Daclatasvir (Table 1) [98].

#### 7.2.4. Darunavir

Darunavir, also known as Prezista, is an anti-retroviral protease inhibitor and is used to treat HIV infections. Darunavir has a similar mechanism of action as Atazanavir, where it inhibits the cleavage of viral Gag and Gag-Pol polyproteins, as well as prevents enzymatic binding, dimerization, and catalytic activity of viral proteases [99]. This drug is currently in Phase 3 and Phase 4 clinical trials for the treatment of SARS-CoV-2 in combination with other drugs in multiple countries worldwide (Table 1) [78,84].

### 7.3. Viral Entry Inhibitor

#### 7.3.1. Hydroxychloroquine

Hydroxychloroquine is an immunosuppressive agent used to treat various autoimmune disorders, such as rheumatoid arthritis, Sjogren’s syndrome, and systemic lupus erythematosus. Hydroxychloroquine is also used as a potent antiparasitic drug and has been FDA-approved to treat malaria since 1955. The proposed antimalarial activity results from the elevation of intravesical pH to a level that inhibits the liposomal activity of antigen-presenting cells, thereby preventing antigen processing and MHC class II-mediated autoantigen presentation to T cells and ultimately inhibiting autophagy. By preventing antigen processing, hydroxychloroquine reduces T cell activation and differentiation [100,101]. Hydroxychloroquine also reduces the release of certain cytokines like IL-1 and TNFa that help to reduce inflammation. The alteration of endosomal pH by hydroxychloroquine also suppresses toll-like receptor (TLR) signaling by interrupting the binding between TLR7 and TLR9 as well as to their respective RNA or DNA ligands. Hydroxychloroquine is also shown to inhibit the terminal glycosylation of the ACE2 receptor, which in turn inhibits SARS-CoV-2 entry, infection, and disease progression [102]. Hydroxychloroquine has also been associated with reducing the risk of thrombosis, one of the major risk-factors in SARS-CoV-2 patients [103]. Although hydroxychloroquine is a major drug used both prophylactically and for the direct treatment of COVID-19 patients, but recently some adverse side-effects were reported, including cardiac arrest and ventricular arrhythmias [104,105,106]. Careful clinical examinations are still underway to validate the effects of hydroxychloroquine on COVID-19 patients. Due to the severity of its progression and the nonavailability of other effective drugs, the FDA gave an accelerated approval for hydroxychloroquine for the treatment of COVID-19 on March 28th, 2020 (Table 1).

#### 7.3.2. Arbidol

Arbidol, also known as Umifenovir, is an indole-based antiviral drug approved for the treatment of the influenza virus in China and Russia, but not currently approved by the FDA in the USA. Arbidol also exerts its antiviral activity against the Zika virus, hepatitis virus, respiratory syncytial virus, coronaviruses MERS-Co-V, and SARS-Co-V [107]. Arbidol predominately inhibits the membrane fusion of the viruses while also decreasing the interaction between the viruses and the host during both endocytosis and exocytosis processes. Additionally, Arbidol interrupts multiple phases of viral cycle replication as a host-targeting agent; from entry, attachment to internalization, and membrane fusion [107,108]. Arbidol is currently under clinical trials for various disease conditions including SARS-CoV-2 (Table 1) [109].

#### 7.3.3. APNO1

APN01 is a human recombinant ACE2 developed for the treatment of pulmonary arterial hypertension, acute lung injury, and acute respiratory distress. As discussed earlier, SARS-CoV-2 enters human cells through the ACE2 receptor. APN01 prevents this ACE2-mediated SARS-CoV-2 interaction, restores the physiological signaling of the ACE2 receptor, and may minimize lung injury and multiple organ dysfunction [110,111]. APN01 is currently under clinical trials in multiple countries for the treatment of novel SARS-CoV-2 (Table 1).

#### 7.3.4. Ivermectin

Ivermectin, also known as Soolantra, Sklice, or Stromectol, is an FDA approved, broad-spectrum, antiviral/antiparasitic drug [112]. Ivermectin selectively binds to glutamate-gated chloride ion channels and increases the permeability of the cell membrane to chloride ions, which in turn causes hyperpolarization of the cell leading to paralysis and parasite death. Ivermectin exhibited antiviral properties against SARS-CoV-2 in vitro through the inhibition of IMPα/β1-mediated nuclear import of viral proteins. Ivermectin is currently under clinical trials for the treatment of SARS-Co-V2 (Table 1) [113,114].

### 7.4. Immune Modulators

#### Interferon-alpha (IFNα-2b)

IFNα-2b is a recombinant interferon alpha-2 protein used as an antiviral and/or antineoplastic drug. IFNα-2b binds to type-1 interferon receptors, leading to the dimerization of JAK1 and JAK2 receptors, that leads to JAK trans-phosphorylation, and phosphorylation of STAT1 and STAT2. Dimerized STAT activates multiple antiviral proteins and immunomodulators [115,116]. IFNα-2b also inhibits viral replication, viral proteases, increases immunomodulating activities such as phagocytic activity of the macrophages, and augmentation of the specific cytotoxicity of lymphocytes for target cells. IFNα-2b is approved by the FDA for the treatment of malignant melanomas, hairy cell leukemia, follicular lymphoma, condylomata acuminata, AIDS-related Kaposi’s sarcoma, as well as chronic hepatitis B and C. Based on its antiviral properties, IFNα-2b could be a potential therapeutic compound to treat novel SARS-CoV-2 and is currently under clinical trials (Table 1) [117].

### 7.5. Monoclonal Antibodies

#### 7.5.1. Sarilumab

Sarilumab, also known as Kevzara, is a human monoclonal antibody that blocks the IL-6 receptor. Sarilumab is approved by the FDA for the treatment of rheumatoid arthritis and is currently under clinical trials for the treatment of critically ill COVID-19 patients with pneumonia, either alone or in combination with hydroxychloroquine, azithromycin, and/or corticosteroids (Table 1) [111,118].

#### 7.5.2. Tocilizumab

Tocilizumab, also known as Actemra, is an immunosuppressive drug mainly used for the treatment of rheumatoid arthritis and juvenile idiopathic arthritis. Tocilizumab is a humanized monoclonal antibody that binds both soluble and membrane bound IL-6 receptors, and inhibits the IL-6 signaling pathway [119]. The SARS-CoV-2 virus binds to alveolar epithelial cells and activates both the innate and adaptive immune system, which leads to the release of a vast number of cytokines including IL-6, IL-10, and IL-23. Among these cytokines, IL-6 acts as both a proinflammatory and anti-inflammatory cytokine, while also being present at high-levels for specific autoimmune diseases such as rheumatoid arthritis [120,121,122]. Additionally, IL-6 is found to be one of the most important cytokines involved in COVID-19 disease-mediated inflammatory conditions [123]. IL-6 increases vascular permeability which then allows a large number of bodily fluids and blood cells to enter into lung alveoli, ultimately leading to dyspnea and respiratory failure [124,125]. Tocilizumab has exhibited promising results for critically ill COVID-19 patients with pneumonia and is currently under clinical trial evaluation either alone or in combination with hydroxychloroquine, methylprednisone, or azithromycin for the treatment of SARS-CoV-2 patients (Table 1).

### 7.6. Janus Kinase Inhibitors

#### 7.6.1. Fedratinib

Fedratinib is a selective JAK2 inhibitor approved by FDA for the treatment of myelofibrosis. Several symptoms of COVID-19, such as pulmonary edema and lung failure, liver, heart, and kidney damage, are associated with cytokine storm, manifesting elevated serum levels of IL-17 [37,126]. Both IL-6 and IL-23 activate STAT3 through JAK2, which then promotes IL-17 expression [126,127]. Fedratinib inhibits JAK2 and in turn reduces expression of the inflammatory cytokine IL-17, ultimately reducing cytokine storm-mediated symptoms in critically ill COVID-19 patients. Fedratinib can be used in combination with other antiviral drugs and supportive treatments but cannot be used alone because JAK2 inhibition is reversible [126,128].

#### 7.6.2. Baricitinib

Baricitinib is a selective and reversible inhibitor of both JAK1 and JAK2 which is approved for the treatment of rheumatoid arthritis. Baricitinib has the same mechanism of action as Fedratinib, as it prevents the release of proinflammatory cytokines. Baricitinib can be used in combination with other antiviral drugs as well as with supportive treatment [96,129,130,131]. Baricitinib is currently in clinical trials for the treatment of SARS-CoV-2 (Table 1).

### 7.7. Nutritional Supplements

#### 7.7.1. Vitamin C

Vitamin C, also known as ascorbic acid, plays a vital role in multiple physiological responses in the human body. T-lymphocytes and NK cells play an important role in the immune response against viral infections, inhibiting reactive oxygen species production, and remodulating the cytokine network in systemic inflammatory syndrome. Vitamin C boosts immunity by stimulating IFN production, stimulating lymphocyte proliferation, and enhancing the neutrophil phagocytic capability [132,133,134,135]. Vitamin C is currently under clinical trials for reducing mortality in critically ill patients infected with SARS-CoV-2 (Table 1).

#### 7.7.2. Vitamin D

Vitamin D, a hormone produced by human body by using sunlight, plays an important role in adaptive immunity, as well as immune cell differentiation, proliferation, and maturation. Due to these properties, vitamin D is currently in clinical trials as an immune modulator for the treatment of novel SARS-CoV-2 (Table 1) [136,137,138].

#### 7.7.3. Folic Acid

Folic acid, a member of the vitamin B family (B9), is an essential molecule for the synthesis of purines, pyrimidines, and methionine for RNA, DNA, and protein synthesis. Folic acid cannot be synthesized in the human body, but it is important for rapid cell proliferation [139]. Folic acid is currently in Phase 2 and Phase 3 clinical trials in combination with vitamin C, azithromycin, and hydroxychloroquine sulfate, for the treatment of high-risk novel SARS-CoV-2 patients (Table 1) [96,111].

### 7.8. Miscellaneous

#### 7.8.1. Telbivudine

Telbivudine is an antiviral thymidine nucleoside analog with activity against the hepatitis B virus DNA polymerase. It is also an FDA-approved for the treatment of chronic hepatitis B. Telbivudine undergoes phosphorylation by cellular kinases yielding its active form, Telbivudine 5′-triphosphate, which competitively inhibits thymidine 5′-triphosphate for incorporation into viral DNA and in turn prevents DNA polymerase activity and causes DNA chain termination [96]. Telbivudine is currently under clinical trials for the treatment of chronic kidney disease, liver cirrhosis, transplantation disorders, and recently for SARS-CoV-2 [111].

#### 7.8.2. Emtricitabine

Emtricitabine, also known as Emtriva, is an antiviral compound used for the treatment of HIV. Emtricitabine is a pro-drug which is converted to emtricitabine 5′-triphosphate upon phosphorylation. Emtricitabine 5′-triphosphate acts as a cytidine analog which inhibits HIV-1 reverse transcriptase and in turn blocks viral DNA synthesis. Emtricitabine is currently under clinical trials for the treatment of the SARS-CoV-2 infection (Table 1) [96,111].

#### 7.8.3. Azithromycin

Azithromycin, an antibiotic, is used to treat a wide range of bacterial infections, including the Streptococcus family, Chlamydia, and Gonorrhea infections. Azithromycin disrupts bacterial growth by interfering with bacterial protein synthesis as well as the translation of mRNA. It is currently under clinical trials for the treatment of novel SARS-CoV-2 (Table 1) [140,141].

#### 7.8.4. Colchicine

Colchicine, an anti-inflammatory compound is used to treat or prevent gout attacks. Additionally, it has antiviral properties against the Flaviviridae virus, and it prevents/reduces viral replication by inhibiting microtubule formation. Colchicine is currently under clinical trials for the treatment of novel SARS-CoV-2 [142,143] (Table 1).

#### 7.8.5. Methylprednisolone

Methylprednisolone is a corticosteroid-based anti-inflammatory drug which is used to treat inflammatory diseases, such as lupus, arthritis, psoriasis, and ulcerative colitis. Methylprednisolone inhibits phospholipase A2 activity which in turn prevents the formation of arachidonic acid while also inhibiting the activation of NF-kB as well as other inflammatory transcription factors [144]. Methylprednisolone is currently under Phase 2/Phase 3 clinical trials for the treatment of critically ill SARS-CoV-2 patients (Table 1) [96,111].

#### 7.8.6. Naproxen

Naproxen exhibits both anti-inflammatory and antiviral properties against the Influenza A virus. Naproxen is also a nonselective COX inhibitor that decreases prostaglandin synthesis as well as inflammatory mediators in SARS-Co-V2 induced inflammation. For these reasons, Naproxen is in clinical trials for the treatment of SARS-CoV-2 patients (Table 1) [96,111,145].

#### 7.8.7. Tilorone

Tilorone, also known as Amixin or Lavomax, is an antiviral drug. Tilorone is an orally active interferon inducer which is approved for the treatment of influenza, acute respiratory viral infection, viral hepatitis, and viral encephalitis in Russia. Tilorone targets the retinoic acid-inducible gene-I-like receptor signaling pathway, which can recognize intracellular viral RNA and induce a cellular response to induce the expression of interferons. It is currently under investigation for the treatment of the Ebola virus and SARS-CoV-2 [111,146,147].

#### 7.8.8. Cobicistat

Cobicistat, also known as Tybost, is primarily used for the treatment of HIV infections in combination with other HIV protease/integrase inhibitors [99]. Cobicistat inhibits the CYP3A-mediated metabolism of other HIV inhibitors and increases their antiviral activity. Cobicistat is currently in clinical trials in combination with antiviral drugs for the treatment of SARS-CoV-2 (Table 1) [111].

#### 7.8.9. Omeprazole

Omeprazole is a proton pump inhibitor used to treat gastroesophageal reflux disease, heartburn, and ulcers. Omeprazole is currently in clinical trials for the treatment of SARS-CoV-2 patients (NCT04333407), although the exact mechanism of how omeprazole inhibits the SARS-CoV-2 infection is unknown (NCT04333407, NCT04527562, NCT04507867) [96,111,148].

#### 7.8.10. Pirfenidone

Pirfenidone is an antifibrotic and anti-inflammatory drug used to treat idiopathic pulmonary fibrosis. Pirfenidone is also known to inhibit the production of collagen as well as fibrogenic mediators such as TGF-b, while it also reduces inflammation by inhibiting the IL-1β and TNF-α [149]. Pirfenidone has shown promising results for the treatment of SARS-CoV-2 pneumonia patients and is currently in Phase 3 clinical trials (Table 1) [149].

#### 7.8.11. Disulfiram

Disulfiram, also known as Antabuse, is a competitive inhibitor of the peripheral benzodiazepine receptor as well as the acetaldehyde dehydrogenase enzyme. The mechanism of action of Disulfiram against SARS-CoV-2 is unknown, However, it has been reported to inhibit the Papain-like protease (PLpro) in MERS and SARS [150]. Disulfiram is currently in Phase 2 clinical trials for the treatment of SARS-CoV-2 infection (Table 1) [111].

#### 7.8.12. Cyclosporin

Cyclosporin is an immunosuppressive drug primarily used for the treatment of organ rejection after transplantation. Cyclosporin is a calcineurin inhibitor which binds to cyclophilin receptors, causing the formation of the cyclosporin-cyclophilin complex, which then also inhibits the calcium-dependent IL-2 pathway in cells [151]. Cyclosporin alone, or in combination with other drugs such as methylprednisone and tacrolimus, is currently in Phase 1, 2, 3, and 4 clinical trials for the treatment of novel coronavirus (Table 1) [96,111].

### 7.9. Convalescent Plasma Therapy

When there is no effective antiviral drug for treating SARS-CoV-2, convalescent plasma therapy has played an important role in treating patients. Convalescent plasma therapy is an adoptive immunotherapy which can be used for treating numerous infectious diseases. Antiviral antibodies from recovered patients can be used to treat other patients with a specific infectious disease and therefore passive immunity can be achieved. Convalescent plasma therapy has exhibited exciting results during SARS, MERS, influenza, and Ebola virus pandemics [152,153]. For SARS-CoV-2, positive patients are treated with the plasma collected from recently recovered SARS-CoV-2 patients [153,154,155,156,157]. SARS-CoV-2 usually peaks during the first week of infection, while symptom onset occurs during the second week. Most patients develop an immune response due to cytokine storm, which can also lead to death. Convalescent plasma therapy may then reduce the serum cytokine response.

In most cases, convalescent plasma therapy is used during the early stages of infection. The effect of convalescent plasma depends upon the patient’s age as well as health conditions, such as comorbidity and stage of illness [153,158,159]. On March 24th, 2020, the FDA issued a specific guideline on exploring the use of convalescent plasma therapy treatment against SARS-CoV-2. Recently, convalescent plasma has been widely recommended as a treatment for COVID-19 (Table 1).

## 8. Vaccine Candidates

The development of an effective SARS-CoV-2 vaccine is essential for preventing the transmission and control of the SARS-CoV-2 pandemic. At this point, it is unclear which vaccine strategy will be the most beneficial, therefore researchers around the world are following numerous strategies to develop an effective vaccine. Recently, mRNA-based vaccines BNT162 developed by Pfizer/BioNTech and mRNA-1273 developed by Moderna Inc. showed around 95% clinical efficacy in Phase 3 safety trials, and these two vaccine candidates are the front runners in a global vaccine race. Recently, BNT162 of Pfizer/BioNTech became the first FDA approved COVID-19 vaccine followed by the mRNA-1273 of Moderna Inc. for vaccination in the USA. The United Kingdom, Bahrain, Canada, and Mexico have also approved the Pfizer/BioNTech COVID-19 vaccine’s emergency use. China and Russia have already approved and are administering CoronaVac and Sputnik V vaccines, respectively, without waiting for the final clinical trials results. According to the WHO, around 162 vaccine candidates are currently in the preclinical evaluation, and 52 are in clinical development. These strategies include inhibition of S protein, proteases, mRNA, RNA-dependent-RNA-polymerase, whole virus vaccines, and antibody vaccines [160,161,162,163]. Some SARS-CoV-2 vaccine candidates that are currently at advanced stage clinical trials are listed below.

### 8.1. mRNA Based Vaccines

Viral RNA vaccines encoding viral antigen have been shown to be both safe and immunogenic in various conditions [164]. mRNA vaccine design includes an open reading frame of the targeted antigen with a 3′ polyadenylated tail. RNA vaccines have a self-adjuvant effect, a short production cycle, high potency, and low manufacturing cost [165].

#### 8.1.1. mRNA-1273

**Figure 3:**
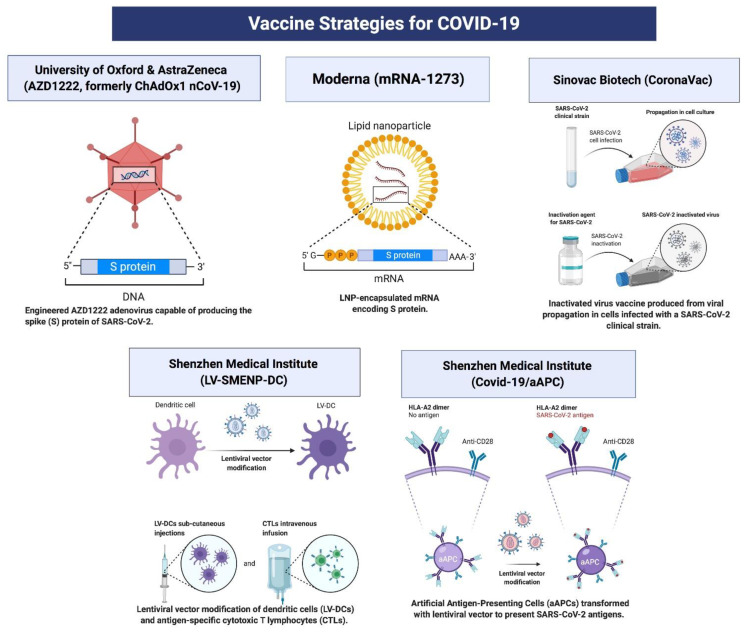
Schematic representation of some ongoing strategies for SARS-CoV-2 vaccine and their respective mechanism of action.

mRNA-1273, a novel lipid nanoparticle encapsulated mRNA-based vaccine, encodes for a full-length, prefusion stabilized spike (S) protein of SARS-CoV-2 (Figure 3). Two proline subunits were included at the top of the central helix in the S2 subunit. This vaccine is developed by the US National Institute of Allergy and Infectious Diseases (NIAID) Vaccine Research Center and Moderna, Inc. [166], and is currently under Phase 3 clinical trials evaluating its efficacy, immunogenicity, and safety (Table 1) [161,167,168]. A recent report from Moderna Inc. claimed to achieve 94.5% efficacy in Phase 3 clinical trials, and recently received FDA emergency approval in the USA. The primary disadvantage of this vaccine is its low half-life, low stability, and it exhibits a 10-fold lower transfection rate than viral vectors [166,169].

#### 8.1.2. BNT162

The BNT162 vaccine is a mRNA-based vaccine, expressing codon-optimized undisclosed SARS-CoV-2 protein(s). It is encapsulated in 80 nm ionizable cationic lipid/phosphatidylcholine/cholesterol or polyethylene glycol lipid nanoparticles developed by BioNTech, Pfizer, and Fosun Pharma. UK became the first county, followed by the USA, Bahrain, Canada, and Mexico to approve the emergency use of BNT162 following a worldwide Phase 3 clinical trial with about 95% efficacy. There are four vaccines listed under this trial (BNT162a1, BNT162b1, BNT162b2, and BNT162c2); each represents a different mRNA format and antigen. Two of the four vaccines are a nucleoside-modified mRNA (modRNA), one uridine containing mRNA (uRNA), and the fourth vaccine is a self-amplifying mRNA (saRNA). The spike (S) protein sequence is also included in two of the four vaccines, while an optimized spike (S) protein receptor-binding domain is included in two other vaccines (Table 1) [160,161,163,167,170]. The main disadvantage of BNT162 includes the need of storage and transportation at extremely cold temperature (below −80 °C) and reduction or loss of vaccine potency within 5 days after thawing. These limitations will pose challenges to transport this vaccine to isolated areas and in developing countries.

#### 8.1.3. LV-SMENP-DC

LV-SMENP-DC is developed by the Shenzhen Geno-Immune Medical Institute, China and is a RNA based vaccine developed by modifying the dendritic cells with lentivirus vectors, manifesting SARS-CoV-2 structural protein domains and the protease using the SMENP minigenes, to express SARS-CoV-2 antigens (Figure 3) [163,171,172]. This vaccine presents antigens to antigen-presenting cells which stimulates cytotoxic T-cells and enhances immunity against SARS-CoV-2. LV-SMENP-DC is currently in Phase 1/2 clinical trials (Table 1) [161,167].

#### 8.1.4. CVnCoV

CVnCoV is developed by CureVac biopharmaceuticals. CVnCoV is mRNA-based nanoparticle vaccine, holds a sequence optimized mRNA coding a stabilized form of spike protein(S) similar to mRNA-1273 and BNT162. The vaccine should be administrated in two doses within 28 days. It is currently in Phase 2/3 clinical trials, no pediatric and pregnancy related efficacy has been established (Table 1) [167].

### 8.2. DNA Based Vaccines

DNA vaccines encode for the antigen and an adjuvant, which induces an adaptive immune response in the host. DNA vaccines are more stable than mRNA vaccines as mRNA is nonintegrating. DNA vaccines also have greater stability, half-life, and immunogenicity when compared to mRNA vaccines. DNA vaccines also played an important role in MERS-CoV and SARS-CoV pandemics earlier [173,174,175,176].

#### 8.2.1. INO-4800

INO-4800 is a DNA plasmid-based vaccine based on the SARS-CoV-2 genetic sequence. It is designed by Inovio Pharmaceuticals to directly deliver optimized plasmids into cells intramuscularly or intradermally using a hand-held smart device called Cellectra. INO-4800 translates into proteins to introduce an immune response inside the host body [174,177]. It is currently in Phase 2/3 clinical trials for determining safety, tolerability, and immunogenicity in up to 6578 healthy individuals across the USA (Table 1) [161,174]. INVO pharmaceuticals had previously developed the most advanced vaccine candidate for MERS-CoV.

#### 8.2.2. GX-19

GX-19, a DNA based vaccine, has been developed by the South Korean-based biotech company Genexine Inc. GX-19 has demonstrated both defensive and resistance efficacy against COVID-19 in primates. GX-19 is administered in two doses on within 28 days and is currently under Phase 2 clinical trials in humans (Table 1) [163,178].

#### 8.2.3. bac-TRL-Spike Vaccine

bac-TRL-Spike is a bacterial vector (*Bifidobacterium longum*) that produces SARS-CoV-2 antigens with its plasmid DNA [179]. Each dose of bac-TRL-Spike contains either 1, 3, or 10 billion colony forming units of live *Bifidobacterium longum* medium. It is engineered to deliver plasmids containing synthetic DNA which encode the spike protein from SARS-CoV-2. It is currently in Phase 1 clinical trials for evaluating its safety, tolerability, and immunogenicity at the University of British Colombia, Canada (Table 1) [161,163,167].

### 8.3. Viral Vector Vaccines

Viral vector vaccines can be recombinant viral vector vaccines or whole virus vaccines. Viral vector vaccines are genetically engineered to reduce the pathogenicity and replication capacity of the virus. The main disadvantages are pre-existing immunity conditions, low-titer production, generation of a replication-competent virus, and the potential for tumorigenicity. Whole-virus vaccines are traditional vaccines which contain the whole virus particle or pathogen that has been weakened, inactivated, or killed to prevent the disease condition [180].

#### 8.3.1. CoronaVac

CoronaVac is an inactivated viral vector vaccine developed by Sinovac Research and Development Co., China, by utilizing the whole inactivated SARS-CoV-2 virus. On August 28th, 2020 China approved the emergency use of CoronaVac to vaccinate high-risk groups such as health care workers. It is currently in Phase 3 clinical trials in Brazil (Figure 3) (Table 1) [167,172,181,182].

#### 8.3.2. ChAdOx1 nCoV-19/AZD-1222

ChAdOx1 nCoV-19, also named as AZD-1222, is a Chimpanzee-based nonreplicating adenovirus vaccine vector (ChAdOx1) that contains the spike (S) protein genetic sequence (Figure 3). Upon vaccination, the spike (S) protein activates the immune system to attack against the SARS-CoV-2, if it infects the host [183,184]. Chimpanzee adenovirus vectors can be given to the patients of 1 week old and up to 90 years of age. However, its main disadvantage is that some people have pre-existing antibodies against this adenovirus (ChAdOx1) vaccine vector. ChAdOx1 nCoV-19 is developed by Oxford University in collaboration with AstraZeneca Inc and can be stored at normal temperature. On December 8th, 2020 manufacture announced that the AZD-1222 has an acceptable safety profile and is efficacious against symptomatic COVID-19, with no hospitalizations or severe reactions. AZD-1222 is currently under Phase 3 clinical trials in the USA, UK, India, and other countries (Table 1) [161,167,183]. Recently, UK and India approved the emergency use of AZD-1222 and started inoculation.

#### 8.3.3. Gam-COVID-Vac Lyo/Sputnik V

Gam-COVID-Vac Lyo, also known as Sputnik V, is developed by the Gamaleya Research Institute of Epidemiology and Microbiology in Russia. It is a two-vector adenovirus vaccine system (Ad5 and Ad26), and both of these adenovirus vectors were previously used to develop other vaccines. The first vaccination contains vector rAD26 with a gene coding for the SARS-CoV-2 S protein. The second vaccination takes place after 21 days, and it contains adenovector AD5 intended to boost long-lasting immunity. Russia approved the first SARS-CoV-2 Vaccine Gam-COVID-Vac Lyo on August 24th, 2020, becoming the first country with an approved vaccine. Gam-COVID-Vac Lyo is currently in Phase 3 clinical trials in the United Arab Emirates, Venezuela, and Phase 2/3 in India (Table 1) [161,163,185].

#### 8.3.4. Coroflu

Coroflu is based on the influenza vaccine candidate M2SR, and is developed by the University of Wisconsin-Madison in collaboration with Bharath Biotech Inc. The genetic sequence of SARS-CoV-2 is inserted into M2SR in order to develop or induce immunity against SARS-CoV-2. M2SR lacks the M2 gene, which obstructs the virus from going through a single round of replication in host cells. Coroflu is currently under preclinical evaluation [161,163,168].

#### 8.3.5. Ad5-nCoV

Ad5-nCoV developed by the Cansino Biologics, China is a genetically engineered replication-defective adenovirus type5 vector vaccine that expresses SARS-CoV-2 spike protein. On 25 June 25 2020, China’s Central Military Commission approved the use of Ad5-nCoV for a year as a “specially needed drug.” It is currently in Phase 3 clinical trials in Russia, Saudi Arabia, and Pakistan [163,186].

#### 8.3.6. COVAXIN (BBV152)

The COVAXIN vaccine was developed by Bharat Biotech, India, in collaboration with the Indian Council of Medical Research and the National Institute of Virology. COVAXIN is an inactive vaccine consisting of SARS-CoV-2 virus particles which were killed so that it will not infect within those injected with it. COVAXIN helps the body to build immunity against the virus and has robust immune response against SARS-CoV-2. It has successfully cleared Phase 1/2 clinical trials and is currently in Phase 3 clinical trials in India (Table 1) [160,161,187,188]. Recently, India approved COVAXIN for emergency use without waiting for the conclusion of Phase 3 clinical trials.

#### 8.3.7. DelNS1-SARS-CoV-2-RBD

DelNS1-SARS-CoV-2-RBD was developed by the University of Hong Kong. This is a live attenuated influenza-based vaccine developed by the deletion of the NS1 gene and reorganized with the RBD domain of the SARS-CoV-2 spike protein on its surface. This vaccine can be used as a nasal spray, and it is currently in preclinical evaluations [160,161,183,187].

### 8.4. Protein Subunit-Based Vaccines

Protein subunit-based vaccines consist of a small or minimal amount of SARS-CoV-2 protein subunits that can boost the immune response in the host, when administered with molecular adjuvants. This approach lies between the inactivated viral vaccine and nuclei acid-based techniques. Protein subunit vaccine candidates also contain recombinant spike protein(S) to be resistant to proteolytic cleavage and capable of binding ACE2 receptors with high affinity [160,189].

#### 8.4.1. NVX-CoV2373

NVX-CoV2373, developed by Novavax, is a SARS-CoV-2 rS nanoparticle vaccine with or without matrix-M-adjuvant. NVX-CoV2373 is developed by recombinant nanoparticle technology that blocks the binding of spike protein to the targeted ACE2 receptor, preventing a crucial step in the viral infection process, enhancing immune response, and stimulating high-levels of neutralizing antibodies. In clinical trials this vaccine induced the development of anti-spike IgG antibodies and wild-type virus-neutralizing antibody responses in humans [188,189,190,191]. Novavax was awarded USD 1.6 billion by the U.S. government as part of Operation Warp Speed (OWS), a U.S. government program to deliver a safe and effective SARS-CoV-2 vaccine. Novartis announced that NVX-CoV2373 showed promising results in Phase 3 clinical trials (Table 1) [160,161,184,187].

#### 8.4.2. SCB-2019

SCB-2019 was developed by Clover Biopharmaceuticals, by using a recombinant Trimer-Tag technology in combination with GSK’s pandemic adjuvant. SCB-2019 is an S-trimer subunit vaccine that resembles the viral trimeric spike protein, and it is produced by a mammalian cell-culture production system. According to preclinical data, the addition of GSK’s pandemic adjuvant to SCB-2019 resulted in high neutralizing antibody levels in multiple animal species. SCB-2019 is currently in Phase 1 clinical trials in Australia (Table 1) [160,161,163,167,187].

#### 8.4.3. PittCoVacc (MNA SARS-CoV-2)

PittCoVacc was developed by the University of Pittsburgh School of Medicine. PittCoVacc is a recombinant SARS-CoV-2 vaccine that involves the administration of spike protein rSARS-CoV-2 S1 and rSARS-CoV-2-S1fRS09 developed by the Micro-Needle Array (MNA)-based technique. MNA SARS-CoV-2 is a skin patch tipped with 400 tiny needles made of sugar and when placed on the skin, the needles dissolve to deliver virus proteins into the body. MNA SARS-CoV-2 is currently in clinical trials for SARS-CoV-2 patients, especially those with oropharyngeal dysphagia (NCT04346212, NCT04375709, NCT04537559) [161,163,187].

#### 8.4.4. Triple Antigen Vaccine

The triple antigen vaccine was developed by Premas Biotech in India. It is a multi-subunit vaccine developed by recombinant technology manifesting three SARS-CoV-2 antigen subunits expressing spike (S), envelope (E), and membrane (M) proteins. This multi-antigenic approach is one of the best methods for developing vaccines, as it can overcome future mutation problems. This vaccine is currently in preclinical investigations in India [161,163,187].

#### 8.4.5. Covid-19 aAPC Vaccine

Covid-19 aAPC is prepared by the transfection of antigen-presenting cells with a genetically modified lentivirus encoding the SARS-CoV-2 structural and protease protein domains (Figure 3). This vaccine is developed by Shenzhen Geno-Immune Medical Institute in China. It is currently in Phase 1 clinical trials for evaluating its safety and immunogenicity (NCT04299724) [161,163,167,181,187].

### 8.5. Inactivated Vaccines

#### BBIBP-CorV

BBIBP-CorV was developed by the Beijing Institute of Biological Products and Sinopharm in China. BBIBP-CorV vaccine contains a SARS-CoV-2 strain inactivated inside Vero Cells. BBIBP-CorV have the ability to induce high levels of neutralizing antibody titers in preclinical data. Phase 3 clinical trials started in the United Arab Emirates in July and showed 86% efficacy. On December 9th^,^ 2020 United Arab Emirates government gave full approval for this vaccine to use for COVID-19 patients [192,193].

### 8.6. Repurposed Vaccines

#### BCG Vaccine

BCG, or Bacilli Calmette-Guerin, is a live vaccine developed for tuberculosis (TB). BCG is currently under clinical trials for healthcare workers as a defense against SARS-CoV-2. In a preliminary analysis, BCG vaccination programs appear to lower infection and mortality rates from SARS-CoV-2. It is currently in Phase 4 clinical trials for the treatment of SARS-CoV-2 (Table 1) [187,194].

## 9. Conclusions

SARS-CoV-2 virus that is also commonly referred as COVID-19 is similar to all other pandemics throughout history, as it infects without discrimination of nationality, religion, creed, and color. However, COVID-19 pandemic is slightly different as it quickly disseminates throughout the world after its initial reporting in Wuhan, China in December 2019, and so far, 213 countries and territories have reported confirmed COVID-19 cases. This quick dissemination is due to the 21st century geopolitical open borders, ease of travel, and also due to the initial negligence of the severity of this virus. International efforts and unanimity are crucial and is the need of the hour to diminish the destructive effects of the COVID-19 pandemic on health care systems, socioeconomic balances, and on the future of some nations.

Due to the recent progress in virology, molecular biology, and pharmacology fields, we were quickly able to dissect and understand the COVID-19 causing virus structure, functions, lifecycle, and pathophysiological characteristics. This leads to several pharmacological intervention approaches for drug repurposing and vaccine development to treat or reduce the severity of patients’ symptoms and death. The tsunamis of COVID-19 related publications and clinical trials show that the scientists and clinicians are not leaving any stone unturned to find a cure for this virus infection, and hope the promising outcome is near in future.

Drug repurposing remains an attractive alternative for developing new drugs for emerging novel healthcare threats such as in the case of COVID-19 outbreak. As per the WHO and COVID-19-NMA project (https://covid-nma.com/dataviz/), there are a total of 2262 treatment studies currently registered worldwide [111,161,163]. Similarly, vaccines typically take about 10 years to develop, and pass through the rigorous preclinical and clinical trials. However, incredibly, within a few weeks of the publication of virus genome sequence, numerous vaccine trials were started and are already in advanced stages of patient trials. Among these vaccines, BNT162, mRNA-1273, and AZD-1222 from Pfizer/BioNTech, Moderna, and AstraZeneca Inc., respectively, have completed the most advanced Phase 3 clinical trials and are the winners in the COVID-19 vaccine race [167,172]. BNT-162 and mRNA-1273 vaccines showed around 95% clinical efficacies in clinical trials, and received FDA emergency use approval in the USA, and now receiving authorizations in other countries as well.

Although these leading COVID-19 vaccine candidates have progressed to advanced stages of clinical development and approvals at exceptional speed, uncertainties related to lack of robust safety clinical data is of public concern. Additionally, scale-up manufacturing, extremely cold temperature transportation, and storage requirements of these vaccines will be a major hurdle in the near future. Another challenge for a successful vaccine is the recent sequencing data that indicate the high mutation rate of SARS-CoV-2, approximately 25 mutations per year [195]. Hopefully, future studies will be able to resolve these questions and come up with effective treatments and vaccines against this deadly virus. In the past two decades three coronaviruses emerged from animal reservoirs, and future outbreaks of similar viruses and pathogens are likely to continue. Therefore, policies and efforts should be made to prevent such outbreaks, and to develop effective common vaccines. Overall, the landscape of therapeutics, diagnostics, and vaccines for COVID-19 is evolving at warp speed with promising results already, which is a significant achievement.

## Figures and Tables

**Table 1:** Therapeutics and vaccines under development for COVID-19.

Drug Name	Clinical Trial	Current Phase	Drug Name	Clinical Trial	Current Phase	Drug Name	Clinical Trial	Current Phase
*RNA-dependent RNA Polymerase inhibitors*								
	NCT04410354	II		NCT04359615	IV		NCT04411433	III
	NCT04431453	II/III		NCT04303299	III	Favipiravir	NCT04499677	II
	NCT04593940	III		NCT04336904	III		NCT04445467	II
	NCT04252664	III		NCT04346628	II			
	NCT04292899	III		NCT04464408	II/III		NCT04402203	II/III
	NCT04409262	III		NCT04558463	III		NCT04494399	II
	NCT04501952	III		NCT04425460	III	Ribavirin	NCT04392427	III
Remdesivir	NCT04257656	III	Favipiravir	NCT04358549	II		NCT04460443	II/III
	NCT04401579	III		NCT04434248	II/III			
	NCT04492475	III		NCT04387760	II		NCT04460443	II/III
	NCT04280705	III		NCT04475991	II		NCT04498936	IV
	NCT04610541	III		NCT04448119	II	Sofosbuvir	NCT04443725	II/III
	NCT04321616	II/III		NCT04346628	II		NCT04561063	II
	NCT04488081	II		NCT04542694	III		NCT04497649	II/III
	NCT04546581	III		NCT04349241	III		NCT04530422	III
	NCT04501978	III		NCT04402203	II/III		NCT04535869	III
	NCT04292730	III		NCT04351295	II/III			
*Viral Protease Inhibitors*								
	NCT04350671	IV		NCT04328012	II/III	Darunavir	NCT04252274	III
	NCT02735707	IV		NCT04403100	III			
	NCT04372628	II		NCT04365582	III			
	NCT04330690	II		NCT04255017	IV		NCT04468087	II/III
Lopinavir/Ritonavir	NCT04346147	II	Lopinavir/Ritonavir	NCT04321174	III	Atazanavir	NCT04452565	II/III
	NCT04499677	II		NCT04466241	II/III		NCT04459286	II
	NCT04409483	III		NCT04364022	III			
	NCT04328285	III		NCT04359095	II/III			
	NCT04286503	IV		NCT04350684	IV			
*Viral entry inhibitors*								
	NCT04382625	IV		NCT04387760	II		NCT04510233	II
	NCT04355026	IV		NCT04391127	III		NCT04381884	II
	NCT04340544	II		NCT04370782	IV		NCT04530474	III
	NCT04364815	III		NCT04397328	III		NCT04523831	III
	NCT04365231	III		NCT04329923	II		NCT04360356	II/III
Hydroxychloroquine	NCT04358081	III	Hydroxychloroquine	NCT04353271	II/III	Ivermectin	NCT04529525	II/III
	NCT04363866	II		NCT04392128	II		NCT04438850	II
	NCT04329832	II		NCT04377646	III		NCT04405843	II/III
	NCT04351620	I		NCT04405921	III		NCT04391127	III
	NCT04361318	II/III		NCT04359537	II		NCT04551755	II
	NCT04429867	IV		NCT04362332	IV		NCT04407130	II
	NCT04329611	III		NCT04435808	I/II		NCT04435587	IV
	NCT04345692	III		NCT04347889	II			
	NCT04328272	III		NCT04359953	III	APNO1	NCT04335136	II
	NCT04385264	II/III		NCT04522466	III		NCT04287686	NA
	NCT04466540	IV		NCT04369742	II			
	NCT04307693	II		NCT04331834	III		NCT04350684	IV
	NCT04371406	III		NCT04336332	II	Arbidol	NCT04286503	IV
	NCT04333225	II		NCT04372017	III		NCT04260594	IV
	NCT04342221	III		NCT04394442	II		NCT04476719	I
	NCT04334382	III		NCT04315896	III			
*Monoclonal antibodies*								
	NCT04315298	II/III		NCT04445272	II		NCT04372186	III
	NCT04341870	II/III		NCT04479358	II		NCT04356937	III
	NCT04357808	II		NCT04317092	II		NCT04320615	III
Sarilumab	NCT04359901	II	Tocilizumab	NCT04345445	III	Tocilizumab	NCT04377503	II
	NCT04357860	II		NCT04435717	II		NCT04363736	II
	NCT04327388	III		NCT04412772	III		NCT04363853	II
	NCT04324073	II/III		NCT04331795	II		NCT04361032	III
	NCT02735707	IV		NCT04377750	IV		NCT04409262	III
				NCT04332094	II		NCT04424056	III
				NCT04377659	II		NCT04335305	II
				NCT04412291	II		NCT04403685	III
				NCT04346355	II		NCT04335071	II
*Nutritional supplements*								
	NCT04395768	II	Vitamin C	NCT04335084	II		NCT04483635	III
	NCT04264533	II		NCT04334967	IV	Vitamin D	NCT04335084	II
Vitamin C	NCT04363216	II					NCT04536298	III
	NCT04347889	II		NCT04483635	III		NCT04385940	III
	NCT04468139	IV	Vitamin D	NCT04552951	IV			
	NCT04401150	III		NCT04535791	III	Folic acid	NCT04354428	II/III
	NCT04357782	I/II		NCT04482673	IV			
	NCT04344184	II		NCT04502667	III			
*Miscellaneous*								
Emtricitabine	NCT04519125	II/III		NCT04354428	II/III		NCT04360980	II
	NCT04405271	III		NCT04334382	III		NCT04392141	I/II
	NCT04334928	III		NCT04358081	III		NCT04375202	II
				NCT04359316	IV	Colchicine	NCT04355143	II
	NCT04341038	III	Azithromycin	NCT04371406	III		NCT04492358	II/III
	NCT04438980	III		NCT04339426	II		NCT04350320	III
	NCT04485429	III		NCT04381962	III		NCT04516941	III
	NCT04499313	III		NCT04332107	III		NCT04472611	III
Methylprednisolone	NCT04377503	II		NCT04359953	III		NCT04326790	II
	NCT04345445	III		NCT04358068	II			
	NCT04355247	II		NCT04332094	II		NCT04252274	III
	NCT04329650	II		NCT04363060	III	Cobicistat	NCT04386447	II
	NCT04263402	IV		NCT04370782	IV		NCT04366089	II
	NCT04528888	III						
						Interferon-alpha	NCT04349410	II/III
	NCT04341038	III		NCT04340232	II/III	(IFNα-2b)	NCT04379518	I/II
Cyclosporin	NCT04392531	IV		NCT04421027	III			
	NCT04540926	I/II		NCT04358614	II/III	Naproxen	NCT04325633	III
	NCT04420364	II/III	Baricitinib	NCT04373044	II			
				NCT04393051	II	Pirfenidone	NCT04282902	III
	NCT04425915	III		NCT04401579	III			
	NCT04345523	II		NCT04321993	II	Disulfiram	NCT04485130	II
Convalescent plasma therapy	NCT04346446	II		NCT04346147	II			
	NCT04403477	II						
	NCT04372979	III						
	NCT04407208	I						
	NCT04558476	II						
*Vaccines*								
	NCT04283461	I	COVAXIN	NCT04641481	III	Gam-COVID-Vac	NCT04437875	I/II
mRNA-1273	NCT04470427	III		NCT04471519	I/II	Lyo		
	NCT04405076	II						
				NCT04368728	II/III		NCT04456595	III
INO-4800	NCT04336410	I	BNT162	NCT04537949	I/II		NCT04551547	I/II
	NCT04447781	I/II		NCT04523571	I	CaronaVac	NCT04383574	I/II
				NCT04380701	I/II		NCT04352608	I/II
LV-SMENP-DC	NCT04276896	I/II					NCT04508075	III
			GX-19	NCT04445389	I/II			
	NCT04368988	I/II					NCT04348370	IV
NVX-CoV2373	NCT04533399	II		NCT04324606	I/II		NCT04328441	III
	NCT04583995	III		NCT04400838	II/III		NCT04362124	III
	NCT04611802	III	ChAdOx1 nCoV-19/ AZD-1222	NCT04540393	III	BCG vaccine	NCT04417335	IV
				NCT04516746	III		NCT04379336	III
bac-TRL-Spike Vaccine	NCT04334980	I		NCT04444674	I/II		NCT04350931	III
				NCT04536051	III		NCT04534803	III
				NCT04568031	I/II		NCT04475302	III
SCB-2019	NCT04405908	I					NCT04327206	III
			Covid-19 aAPC	NCT04299724	I		NCT04537663	IV
